# Special Issue “Recent Advances in Biomaterials and Dental Disease” Part I

**DOI:** 10.3390/bioengineering10010055

**Published:** 2023-01-01

**Authors:** Naji Kharouf, Salvatore Sauro, Louis Hardan, Youssef Haikel, Davide Mancino

**Affiliations:** 1 Department of Biomaterials and Bioengeneering, INSERM UMR_S, Strasbourg University, 67000 Strasbourg, France; 2 Department of Endodontics, Faculty of Dental Medicine, Strasbourg University, 67000 Strasbourg, France; 3 Dental Biomaterials and Minimally Invasive Dentistry, Department of Dentistry, CEU Cardenal Herrera University, CEU Universities, C/Santiago Ramón y Cajal, s/n., Alfara del Patriarca, 46115 Valencia, Spain; 4 Department of Therapeutic Dentistry, I. M. Sechenov First Moscow State Medical University, 119146 Moscow, Russia; 5 Department of Restorative Dentistry, School of Dentistry, Saint-Joseph University, Beirut 1107 2180, Lebanon; 6 Pôle de Médecine et Chirurgie Bucco-Dentaire, Hôpital Civil, Hôpitaux Universitaire de Strasbourg, 67000 Strasbourg, France

## 1. Introduction

**Figure 1:**
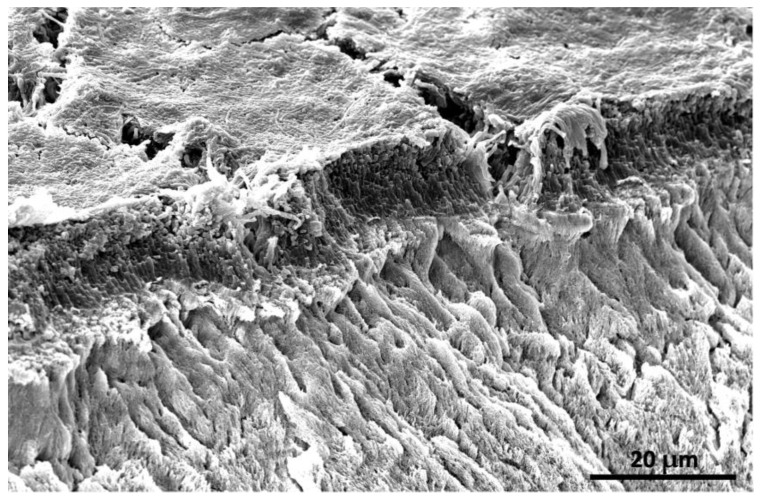
Scanning electron microscopy (SEM) shows the adhesion of oral biofilm to the enamel surface.

Oral cavities provide an entry point for food and nutrients. Teeth consist of various hard and soft tissues, such as enamel, dentin, cementum, dental pulp, and the root canal system. Adsorbed macromolecules which are delivered by bacteria, blood, saliva, gingival fluids, particles, and molecules from the diet comprise the salivary pellicles [1]. Oral bacteria can adhere to the enamel surfaces (Figure 1) and demineralize dental hard tissues, resulting in the development of dental caries.

## 2. Individual Contribution

### 2.1. Enamel and Dentin Restoration

Once caries are established in the enamel and dentin structures, they can be eliminated with burs; then, the enamel and dentin structures can be restored with direct or indirect dental restorations [2,3,4,5]. These restorative biomaterials bond effectively to dental tissues, providing both durability and aesthetically satisfactory results [2,4,6,7]. Different desensitizers can produce chemical interactions with dentinal structures; these can be used in luting cements and may impact their bonding and sealing properties [8].

### 2.2. Modifications of Coronal Restorative Materials

Researchers have been working to improve the biological properties of coronal restorative biomaterials through adding bioactive molecules to their composition in order to ensure antibacterial and antioxidant properties [2,9]. Kharouf et al. [2] used pyrogallol to modify a dental adhesive. They reported that pyrogallol, a polyphenol–vegetable tannin, may preserve the polymer–dentin bonding interface and provide a certain degree of antibacterial activity.

### 2.3. Root Canal Treatment

Once bacteria arrive in the root pulp system, an endodontic treatment should be performed. The choice of endodontic treatment depends on the clinical situation [10].

Vital pulp therapy could be performed using pulp capping, partial pulpotomy, of full pulpotomy therapy [11,12,13]. Successful endodontic treatment consists of an appropriate access cavity [14], good shaping [15], proper cleaning using irrigants [16], and an optimal 3D obturation for the root canal system using sealer and gutta-percha [10,17,18]. Endodontic sealers must be used with gutta-percha to entomb the bacteria and ensure optimal sealing for the root canal system [19]. Several endodontic materials with different chemical compositions, such as zinc oxide eugenol, gutta-percha flow, epoxy–resin, and calcium silicate, have been introduced in the dental market. In addition, different studies have modified endodontic sealers with different polyphenols to enhance their properties [20]. Various endodontic treatments, such as pulpotomy, pulp capping, perforation, open apex, and the retrograde endodontic procedure, should be performed with materials that are more viscous than endodontic sealers, for example, endodontic cements (putty) [21].

### 2.4. Calcium Silicate Materials in Endodontic Treatment

Recently, calcium silicate (CS) materials were introduced to the dental market. The advantageous antibacterial activity, biocompatibility, filling ability, and physicochemical properties of CS materials have led this product to be the primary choice in modern clinical endodontics [11,21,22]. Moreover, these materials can be used in both permanent and primary teeth [23]. Despite the advantages of the evolution which has occurred in the endodontic field, some cases should be retreated with mechanical and chemical procedures to eliminate root canal materials which are no longer in use, clean the root canal system, and retreat re-infected teeth [24,25]. When orthograde endodontic treatment cannot be performed correctly, surgical endodontic treatment must be performed to improve the effectiveness of root canal debridement, and the quality of the retro-preparation and the retro-obturation of the apical part of the dental root [26,27]. Moreover, different regenerative endodontic techniques could offer good alternatives for pulp revitalization in some endodontic situations [28].

### 2.5. Final Restoration Materials and Preparation Steps

Today, a major challenge for dentists is in restoring the functionality of endodontically treated teeth. Several materials and techniques have been proposed to have a good restoration ability; these also ensure the durability of a restoration. Until now, there is no specific restoration for endodontically treated teeth which could avoid the restorative complications that lead to failure and tooth extraction [29,30,31]. Recently, Kharouf et al. [32] studied a new material—a bidirectional, spiral-winding, fiber-reinforced composite—which demonstrated a high compressive strength resistance; this material could be a promising solution for restoring endodontically treated teeth. In addition, different techniques could be used to fabricate the final restoration, such as the digital light-processing 3D-printing technique [7]. Several materials can be used in the final restoration, such as composite resin, zirconia, and lithium disilicate. Cleaning and disinfection procedures of these restorations, including impression materials, are recommended to ensure the quality of dental treatment. Hardan et al. reported that several disinfection agents could be used to disinfect dental impression materials [33]. Harouny et al. demonstrated that the use of phosphoric acid on the lithium disilicate surface provide efficient cleaning [34]. Therefore, the main aim of studies in the dental restoration field is to ameliorate the mechanical properties, bond strength, and durability of direct and indirect restoration approaches, as well as to improve the techniques used to bond brackets to teeth with different restorative materials in orthodontic treatments [35,36].

### 2.6. Dental Implant

Dental implants have been a solution for replacing lost teeth and for supporting fixed or removable prostheses for over 40 years. However, during the implantation planning stage, any sources of infection in the edentulous area should be carefully identified in advance to avoid complications with the dental implants [37].

## 3. Conclusions

Finally, the researchers involved in this Special Issue will continue their studies to improve the existent dental biomaterials in order to have an optimal dental treatment with biocompatible, bioactive, and stable properties.

As the Guest Editors, we sincerely value and thank the reviewers for their insightful comments and the support of the team at *Bioengineering*. Finally, we express our gratitude to all contributing authors for their valuable research. Altogether, the 15 research papers/reviews in this Special Issue—entitled Recent Advances in Biomaterials and Dental Disease—reflect the importance of in vitro and in vivo studies for improving the efficacy of using biomaterials in dental treatment.
